# Microstructural analyses of the posterior cerebellar lobules in relapsing-onset multiple sclerosis and their implication in cognitive impairment

**DOI:** 10.1371/journal.pone.0182479

**Published:** 2017-08-08

**Authors:** Amandine Moroso, Aurélie Ruet, Delphine Lamargue-Hamel, Fanny Munsch, Mathilde Deloire, Pierrick Coupé, Julie Charré-Morin, Aurore Saubusse, Jean-Christophe Ouallet, Vincent Planche, Thomas Tourdias, Vincent Dousset, Bruno Brochet

**Affiliations:** 1 Univ. Bordeaux, Bordeaux, France; 2 CHU de Bordeaux, INSERM-CHU CIC-P 0005, & Services de Neurologie et Neuroradiologie, Bordeaux, France; 3 Neurocentre Magendie, INSERM U1215, Bordeaux, France; 4 LaBRI, UMR 5800, PICTURA, Talence, France; Charite Universitatsmedizin Berlin, GERMANY

## Abstract

**Background:**

The posterior cerebellar lobules seem to be the anatomical substrate of cognitive cerebellar processes, but their microstructural alterations in multiple sclerosis (MS) remain unclear.

**Objectives:**

To correlate diffusion metrics in lobules VI to VIIIb in persons with clinically isolated syndrome (PwCIS) and in cognitively impaired persons with MS (CIPwMS) with their cognitive performances.

**Methods:**

Sixty-nine patients (37 PwCIS, 32 CIPwMS) and 36 matched healthy subjects (HS) underwent 3T magnetic resonance imaging, including 3D T1-weighted and diffusion tensor imaging (DTI). Fractional anisotropy (FA) and mean diffusivity (MD) were calculated within each lobule and in the cerebellar peduncles. We investigated the correlations between cognitive outcomes and the diffusion parameters of cerebellar sub-structures and performed multiple linear regression analysis to predict cognitive disability.

**Results:**

FA was generally lower and MD was higher in the cerebellum and specifically in the vermis Crus II, lobules VIIb and VIIIb in CIPwMS compared with PwCIS and HS. In hierarchical regression analyses, 31% of the working memory z score variance was explained by FA in the left lobule VI and in the left superior peduncle. Working memory was also associated with MD in the vermis Crus II. FA in the left lobule VI and right VIIIa predicted part of the information processing speed (IPS) z scores.

**Conclusion:**

DTI indicators of cerebellar microstructural damage were associated with cognitive deficits in MS. Our results suggested that cerebellar lobular alterations have an impact on attention, working memory and IPS.

## Introduction

Diffusion tensor imaging (DTI) is a sensitive method for studying microstructural changes in the brain [1]. It has been used in recent years in several studies to obtain a better understanding of the cognitive impairment associated with multiple sclerosis (CIAMS) [2]. CIAMS is common and can affect persons with multiple sclerosis (PwMS) at all stages of the disease, including the early stages, such as clinically isolated syndrome (CIS) [3]. CIAMS implies several cognitive domains including episodic memory, attention, working memory and executive functions [3]. However, the slowness of the information processing speed (IPS) is the main cognitive dysfunction observed in MS even at the earlier stages and is associated with poor prognosis, significant consequences on employment status and decreased quality of life [4]. The pathogenic mechanisms underlying CIAMS are still not fully understood [2,3]. Magnetic resonance imaging (MRI) studies suggested that diffuse damage of the cerebral white matter affecting important cognitive networks [5,6] could play a role in the early stages, but a role for the involvement of grey matter (GM), including the thalami, has also been demonstrated [7]. It is now established that the cerebellum plays an important role in cognition in general [8]. Schmahmann *et al*. suggested that the cerebellum regulate speed, consistency and accuracy of cognitive processes. The cerebellum is supposed to integrate and permit cognitive facilitation and optimisation in order to obtain automation. Then, cerebellar damage could result in a « dysmetria of thought » defined by analogy with motor dysmetria [8,9]. In MS, cerebellar dysfunction is associated with cognitive deficits [10,11], particularly IPS [12]. MS is associated with cerebellar damage, and extensive demyelination has been observed in the cerebellar cortex [13]. An association between CIAMS and cerebellar GM atrophy [14,15] and lesion volume [11,16] has been reported. Several DTI studies of the brain reported abnormal DTI metrics such as fractional anisotropy (FA) and mean diffusivity (MD) associated with CIAMS in the cerebellar peduncles or the cerebellar parenchyma. This association suggests an anatomical disconnection between the cerebral associative areas and the cerebellum [17–22]. Functional magnetic resonance imaging (fMRI) confirmed the existence of a functional cortico-cerebellar disconnection associated with CIAMS [23] and provided evidence of the cognitive specificity of posterior cerebellar lobules in healthy subjects (HS) [24,25]. Posterior lobules integration within the cortico-cerebellar loop has been shown anatomically. Cortico-pontine projections have been evidenced using viral transynaptic tracers in rhesus monkeys showing connexion between hemispheric parts of Crus II and vermian parts of lobules VII et IX on the one hand and area 46 and 9 of the dorsolateral prefrontal and area 5 and 7 of posterior parietal cortices on the other hand [26,27]. A specific cognitive cartography of the posterior cerebellar lobules has been described based on fMRI studies made in the last twenty years [25]. Indeed, in healthy subjects (HS), although all posterior lobules are engaged, some preferential contribution of specific lobules in cognitive domains have been observed, such as the left supero-posterior cerebellum for attention [28], vermis VI and Crus I for verbal working memory and lobule VI, Crus I/II and VIIb for executive functions [24,25].

However, the association between specific alterations of the posterior lobule microstructure and specific cognitive outcomes has not been studied in CIS and MS. Our aim was to study the diffusion metrics in lobules VI to VIIIb in persons with CIS (PwCIS) according to the cognitive status, in PwMS with cognitive impairment (CIPwMS) and in HS as negative controls.

## Methods

### Subjects

Sixty-nine patients (PwCIS and CIPwMS) and 69 HS matched for age, sex and educational level were recruited from June 2010 to December 2014 at the Bordeaux University Hospital Center, France. Out of 69 HS, 36 underwent an MRI scan and all 69 were evaluated with cognitive testing.

All PwCIS (n = 37) were included within 6 months after their first neurological episode and presented at least two asymptomatic cerebral lesions larger than 3 mm on fast fluid-attenuated inversion-recovery (FLAIR) images. For CIPwMS (n = 32) the inclusion criteria were as follows: MS diagnosis according to McDonald’s criteria [29], disease duration >6 months and ≤15 years and mild cognitive impairment defined as two scores beyond one standard deviation (SD) among a large neuropsychological battery. MS patients were treated according to current standards of clinical care.

Exclusion criteria were as follows: age under 18 or over 55 years, history of other neurological or psychiatric disorders, inability to perform computerised tasks or MRI, MS attack in the two months preceding the screening, corticosteroid pulse therapy within two months preceding the screening, severe cognitive deficits (Mini-Mental State Examination <27), and depression (Beck Depression Inventory score (BDI) >27).

Expanded Disability Status Scale (EDSS) score was determined by expert neurologists.

### Standard protocol, approvals, registration, and patient consents

Each subject provided written informed consent. Patients were included from two different studies (REACTIV, ClinicalTrials.gov Identifier: NCT01207856, study concerning cognitively impaired PwCIAMS, and SCI-COG, ClinicalTrials.gov Identifier: NCT01865357, analysing cognitive impairment in patients after a CIS). Both studies were approved by the local ethics committee which is called Comité de Protection des Personnes, Bordeaux.

### Neuropsychological assessment

Neuropsychological evaluation assessed attention, working memory, executive functions and IPS. Because PwCIS and PwCIAMS were included in two different studies, some tests for working memory and verbal fluency differed between the two samples and z scores for cognitive domains were calculated by comparisons with scores obtained in the control group. Attention IPS and executive function tests, other than verbal fluency, were identical for all patients and HS and have been described previously [30]. All patients and their matched HS included in a given study (respectively SCI-COG and REACTIV) were assessed by the same tests.

Each domain was evaluated with the following tests (CIS and matched HS: *; MS and matched HS: **):

1. *Attention*: Test of Attentional Performance (TAP)*^/^** consists of subtests for visual scanning (accurate answers) and visual and auditory divided attention For divided attention, the number of accurate answers and reaction time ratios of the double task (auditory and visual divided attention) to the simple task (auditory or visual divided attention) were considered.
2. Working Memory: Numerical span test (forward*^/^** and backward*^/^**) and Paced-Auditory Serial Addition Test–3 seconds (PASAT)* or working memory subtest of the TAP**.
3. *Executive functions*: Stroop test*^/^** (using the difference between the denomination part and the inhibition task scores) and Word List Generation test (verbal fluency assessment) * or semantic verbal fluency (using animal category) **.
4. *IPS*: Symbol Digit Modalities Test (SDMT)*/**

Depression and anxiety symptoms were measured using Beck’s Depression Inventory (BDI) and State-Trait Anxiety Inventory for adults, subtest State (STAI-S), respectively in all participants.

### MRI acquisition

MRI scans were performed on a 3T Achieva TX system (Philips Healthcare, Best, The Netherlands) with an 8-channel coil. The morphological protocol consisted of 3D T1 weighted MR images acquired using the magnetization prepared rapid gradient echo (MPRAGE) imaging (TR = 8.20 ms, TE = 3.5 ms, TI = 982 ms, α = 7°, FOV = 256 mm, voxel size = 1 mm^3^, 180 slices) and Fast Fluid-Attenuated Inversion-Recovery (FLAIR) images (TR = 11000 ms, TE = 140 ms, TI = 2800 ms, FOV = 230 mm, matrix = 325X352, 45 axial slices, 3-mm thick). The single-shot diffusion weighted EPI DTI sequence included 20 directions (TE = 60 ms, TR = 11676 ms, matrix = 144X144, 75 slices, 1.6-mm thickness, b = 1000 s/mm^2^).

### Image processing and analysis

#### MRI processing

T1-weighted MRI images were processed using the pipeline of the volBrain system (http://volbrain.upv.es). This preprocessing pipeline consisted of a denoising step [31] and an affine registration [32] into the Montreal Neurological Institute (MNI) space.

#### DTI processing

Diffusion MRI images were processed using an in-house pipeline (dtiBrain). First, diffusion-weighted images were denoised [33] to improve the signal-to-noise ratio. Head displacement and distortions induced by eddy currents were then corrected by performing affine registration followed by non-linear registration of all diffusion-weighted images to the b0 image. The direction table was updated with the estimated registration matrices. A non-rigid registration of diffusion-weighted images to the subject’s T1-weighted images in the MNI space was used to compensate for EPI distortions. Finally, a diffusion tensor model was fit at each voxel using FSL 5.0.3 (http://fsl.fmrib.ox.ac.uk/fsl) to estimate the fractional anisotropy (FA) and mean diffusivity (MD) maps.

#### Regions of interest

**Fig 1:**
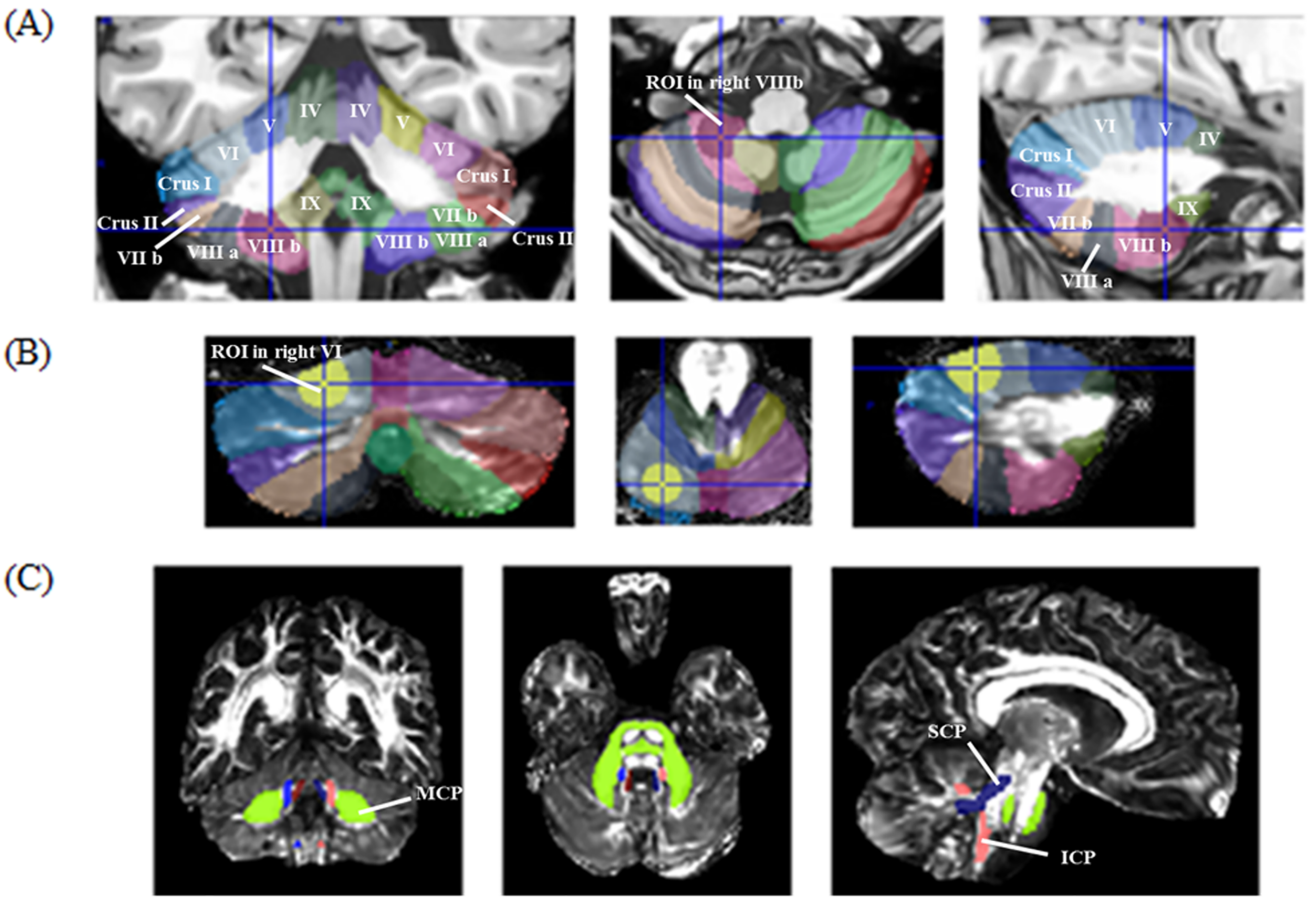
A: Detailed image processing used to obtain lobular and peduncular diffusity data. Cropping and non-linear registration of T1w images over the SUIT template and ROI delineation. B: Registration of the diffusivity map using the transformation estimated with the T1w, diffusivity estimation into ROI. C: Non-linear registration of diffusivity maps and estimation within ROI determined by the JHU-ICBM-DTI-81 WM atlas (FSL).

- **Lobule segmentation**: The spatially unbiased atlas template of the cerebellum and brainstem (SUIT) toolbox 3.0 (SPM 8) was used for cerebellar lobule segmentation [34–36]. The software enables the standardization of lobule size to drive a reliable segmentation by capturing inter-individual variability. First, cerebellums were isolated from brains on 3D T1w in the MNI space, and a non-linear registration of the cropped T1w images over the SUIT template using Dartel was performed [37]. FA and MD maps in the MNI space were transformed into the SUIT space using the deformation field estimated on the T1w images. Moreover, dedicated regions of interest (ROI) were defined in each cerebellar lobule from VI to VIIIb to avoid susceptibility artefacts at the border of the posteriorly located lobules that could artificially modify diffusivity parameters. To define these ROIs, spheres with a radius of 8 mm, adapted to the lobular anatomy, were manually drawn on SUIT cerebellum atlas labels using MRIcron, version 4.8.2014. ROIs were positioned inside the inner part of the posterior lobules in order to minimize CSF partial volume and outer layer artefacts and were replicated for each patient’s lobules to obtain a reproducible and representative sample of FA and MD values per lobule (Fig 1B).
- **Peduncle segmentation**: Finally, because the SUIT toolbox did not include cerebellar peduncle masks, a dedicated pipeline was developed to segment these structures. To do that, we registered FA and MD maps in the MNI space by using affine and non-linear registration (FNIRT, FSL 5.0) with the JHU-ICBM-FA-1mm template as a reference. Then, we used the JHU ICM DTI 81 WM atlas to create binarized masks for each cerebellar peduncle (superior cerebellar peduncle, SCP; medium cerebellar peduncle, MCP; inferior cerebellar peduncle, ICP). Finally, the binarized masks were warped on the FA and MD maps to calculate mean values for each cerebellar peduncle. Therefore, diffusion metrics of superior (SCP), medium (MCP) and inferior cerebellar peduncles (ICP) were independently estimated within ROI determined by the JHU-ICBM-DTI-81 WM atlas (Fig 1C).

The average values of the diffusivity parameters were then calculated in each ROI in cerebellar lobules and peduncles.

### Statistical analyses

All data were analysed with the R package ‘stats’ (version 3.1.3). The normal distribution was tested for all variables with the Shapiro-Wilk test.

Sex and educational level were compared using Chi-square tests. Quantitative clinical and imaging data were compared between PwCIS, PwCIAMS and HS with ANOVA or Kruskal-Wallis tests depending on their distributions. For post hoc analyses, Tukey’s or Nemenyi tests were used to compare two subgroups when ANOVA or Kruskal-Wallis tests showed significant results.

Z scores were calculated according to the formula: (^patient's raw score–HS mean score^ / _HS standard deviation (SD)_) from a population of 69 HS. When a cognitive domain was composed of multiple tests, the mean z score of each test was considered. Cognitive impairment was defined with z scores below -1.5 in a domain. Z score comparisons between PwCIAMS and PwCIS were obtained with the t-test (or Mann-Whitney U tests). A significance threshold of 0.05 was applied.

According to the variable distribution, Spearman or Pearson’s correlations between imaging and cognitive outcome in all patients (CIS and MS) were used. Bonferroni correction for multiple comparisons was applied (p<0.002).

Linear regression analyses were used to predict cognitive outcome, including three hierarchical blocks: 1) clinical data, 2) lobular data, and 3) peduncle data. Each cognitive domain was studied in an independent model. Two prediction models were defined (FA and MD models). The dependent variable and residual normal distributions were checked using the Shapiro test and histograms. Independent variables were entered in the models only if the p value was below 0.10 in univariate analyses.

## Results

### Demographic, clinical data and cognitive assessment

We included 37 PwCIS, 32 CIPwMS and 69 HS. There were no differences for sex, median age and educational level between groups, either when considering the whole HS group or only the HS subgroup that underwent MRI. Table 1 describes the population demographics and clinical characteristics. The mean attention, working memory and IPS z scores were significantly decreased in CIPwMS versus PwCIS. No differences were detected for executive functions. Table 1 shows the z scores for the different cognitive domains for the two groups. IPS slowness occurred in 13.51% of PwCIS and in 71.77% of CIPwMS. Depression scores were higher in CIS (BDI median score = 9 [0–26]; p<0.001) and MS (14 [0–26]; p<0.001) compared to HS (4 [0–23]). Anxiety scores were increased in PwCIS (STAI-S median score = 34 [20–64]; p<0.001) but not CIPwMS compared to HS. No correlation was found between cognitive assessment and anxiety or depression scales.

**Table 1:** Characteristics of the population.

	HS (n = 69)	CIS (n = 37)		MS (n = 32)	
Median age (range) ^a^	36 (21–60)	36 (19–59)^ns^		42 (29–50) ^ns^	
Sex: % of women^b^	50 (72.46%)	29 (78.38%) ^ns^		23 (71.88%)^ns^	
Median EDSS (range)^b^	.	1.00 (0–6)		3.00 (0–8)***	
Mean disease duration (SD) in months^c^	.	4.25 (± 1.98)		106.11 (± 61.44)***	
Median MSSS (range)^c^	.	2.44 (0.67–9.74)		3.55 (0.21–9.09)***	
Educational level^b^	54 (78.26%)	25 (67.57%) ^ns^		21 (65.62%)^ns^	
Mean attention z score (SD)/ % impaired^c^	.	-0.13 (± 0.44)	0	-0.33 (± 0.70)*	6.25
Mean working memory z score (SD)/ % impaired^c^	.	-0.32 (± 0.73)	5.41	-0.97 (± 0.78)**	25
Mean EF z score (SD)/ % impaired^c^	.	-0.54 (± 0.69)	8.11	-0.75 (± 0.83) ^ns^	15.3
Mean IPS z score (SD)/ % impaired^c^	.	-0.57 (±1.06)	13.51	-2.11 (± 1.02)***	71.77

^a^: Results for ANOVA between HS, CIS and MS;

^b^: Results for Fisher’s exact test;

^c^: Results for t tests between CIS and MS patients.

Differences between groups:

^ns^: not significant; p>0.05;

*: p≤0.05;

**: p≤0.01;

***: p≤0.001

EF: Executive functions, IPS: Information Processing Speed

### DTI metrics analyses

Significant differences in the DTI metrics between groups are described in Tables 2 and 3.

**Table 2:** Comparisons of FA between CIS, MS and HC.

	HS (n = 36)	CIS (n = 37)	MS (n = 32)
	Mean ± SD	Mean ± SD	Mean ± SD
	Median (range) ¤	Median (range) ¤	Median (range) ¤
Left VI	0.23 ± 0.03	0.23^c^** ± 0.03	0.20^b^** ± 0.03
Vermis VI	0.12 ± 0.02	0.14^a^** ^c^*** ± 0.02	0.12 ± 0.02
Right VI	0.19 ± 0.03	0.19^c^* ± 0.03	0.17 ± 0.04
Left Crus I	0.23 ± 0.04	0.23^c^* ± 0.04	0.21^b^* ± 0.03
Right Crus I	0.19 ± 0.03	0.19^c^** ± 0.03	0.16^b^* ± 0.03
Vermis VIIb ¤	0.16 (0.11–0.31)	0.17^c^** (0.11–0.26)	0.15 (0.11–0.23)
Right VIIb	0.18 ± 0.03	0.20^c^* ± 0.02	0.17 ± 0.03
Vermis VIIIa	0.17 ± 0.03	0.19^c^* ± 0.04	0.17 ± 0.02
Right VIIIa	0.18 ± 0.03	0.20^a^* ± 0.04	0.18 ± 0.03
Right VIIIb	0.16 ± 0.03	0.18^c^* ± 0.04	0.16 ± 0.04
SCPL	0.55 ± 0.03	0.55^c^*** ± 0.03	0.52^b^** ± 0.03
SCPR ¤	0.55 (0.46–0.59)	0.53^c^** (0.49–0.63)	0.52^b^** (0.42–0.56)
MCP	0.48 ± 0.02	0.48^c^*** ± 0.02	0.46^b^*** ± 0.03
ICPL	0.47 ± 0.03	0.48^c^*** ± 0.03	0.45^b^* ± 0.04
ICPR	0.47 ± 0.03	0.46^c^* ± 0.03	0.44^b^** ± 0.03)

^a^. Comparison between CIS and HC;

^b^. Comparison between MS and HC;

^c^. Comparison between CIS and MS

Differences between groups:

*: p≤0.05;

**: p≤0.01;

***: p≤0.001

^¤^ Kruskal-Wallis test used because of non-linear distribution

SCPL: Superior cerebellar peduncle, left and right (SCPR); MCP: Medium cerebellar peduncle; ICPL: Inferior cerebellar peduncle, left and right (SCPR)

**Table 3:** Comparisons in MD between CIS, MS and HC.

	HS (n = 36)	CIS (n = 37)	MS (n = 32)
	Mean ± SD	Mean ± SD	Mean ± SD
	Median (range) ¤	Median (range) ¤	Median (range) ¤
Vermis Crus II	1.21E-03 ± 8.91E-05	1.18E-03^b^*** ± 1.21E-04	1.30E-03^a^** ± 1.65E-04
Left VIIb ¤	1.08E-03 (8.93E-04–1.61E-03)	1.07E-03^b^* (8.82E-04–1.36E-03)	1.13E-03^a^* (7.61E-04–1.50E-03)
Left VIIIb	1.22E-03 ± 1.83E-04	1.27E-03 ± 1.92E-04	1.40E-03^a^* ± 2.70E-04
Vermis VIIIb	1.13E-03 ± 1.29E-4	1.11E-03^b^* ± 1.24E-04	1.20E-03 ± 1.56E-04
Right VIIIb ¤	1.06E-03 (6.42E-04–1.24E-03)	1.07E-03 (8.37E-04–1.31E-03)	1.13E-03^a^* (8.80E-04–1.38E-03)
SCPL	1.42E-03 ± 9.01E-05	1.40E-03^b^*** ± 9.05E-05	1.54E-03^a^*** ± 1.67E-04
SCPR	1.42E-03 ± 1.04E-04	1.40E-03^b^*** ± 9.49E-05	1.53E-03^a^** ± 1.91E-04
MCP	1.06E-03 ± 3.96E-05	1.06E-03^b^*** ± 3.46E-05	1.11E-03^a^*** ± 6.27E-05
ICPL ¤	1.03E-03 (9.38E-04–1.13E-03)	1.03E-03^b^* (9.57E-04–1.13E-03)	1.08E-03^a^** (1.01E-03–1.27E-03)
ICPR	1.02E-03 ± 3.02E-05	1.03E-03^b^** ± 3.09E-05	1.07E-03^a^*** ± 4.82E-05

^a^ Comparison between MS and HC;

^b^. Comparison between CIS and MS

Differences between groups:

*: p≤0.05;

**: p≤0.01;

***: p≤0.001

^¤^ Kruskal-Wallis test used because of non-linear distribution

SCPL: superior cerebellar peduncle, left, SCPR: superior cerebellar peduncle, right (SCPR); MCP: medium cerebellar peduncle; ICPL: inferior cerebellar peduncle, left; SCPR: inferior cerebellar peduncle, right

No significant differences were found regarding diffusivity metrics between PwCIS and HS.

FA was reduced in a majority of cerebellar substructures in MS compared to CIS and to HS, especially on the right side (Table 2). CIS patients tended to display slightly higher FA values, compared to HS, but this usually did not reach statistical significance. MD was increased in the vermis Crus II, Left VIIb, lobules VIIIb and cerebellar peduncles in CIPwMS compared to HS and to PwCIS.

### Correlations between cognitive outcome and imaging data

Correlations between diffusion metrics and cognitive outcomes are listed in Tables 4 and 5. For the entire patient group, working memory was positively correlated with the FA in the superior cerebellar peduncles, whereas the IPS was positively correlated with FA in the Left VI, Right VIIIa and right VIIIb, and with MD in the vermis Crus II.

**Table 4:** Correlations between FA and cognitive outcome.

	Patients (n = 69)
Working memory	
SCPL	0.54***
SCPR	0.47***
IPS	
Left VI	0.47***
Right VIIIa	0.42**
Right VIIIb	0.43**
MCP	0.47***
ICPL	0.39*

Differences between groups:

*: p≤0.05;

**: p≤0.01;

***: p≤0.001

**Table 5:** Correlations between MD and cognitive outcome.

	Patients (n = 69)
IPS	
Vermis Crus II	-0.45**
SCPL	-0.41**
MCP	-0.38*

SCPL: Superior cerebellar peduncle, left and right (SCPR); MCP: Medium cerebellar peduncle; ICPL: Inferior cerebellar peduncle, left and right (SCPR); Differences between groups:

*: p≤0.05;

**: p≤0.01

The results from multiple hierarchical regression analyses are presented in Tables 6 and 7. FA in the left lobule VI and in the left superior peduncle explained 31% of the variance in the working memory z score. Working memory was also associated with MD in the vermis Crus II. FA in the left VI and right VIIIa also predicted some of the IPS z scores. The microstructure of cerebellar peduncles had an impact on cognitive outcome in almost all tested domains.

**Table 6:** Multivariate analysis model 1 –Cognitive prediction and FA analysis.

	Estimate (B)	Standard Error	P value	Adjusted R2
Attention				
				None
Working Memory				
Intercept	-8.43	1.47	3.39E-7	0.31
Left VI	5.63	2.75	0.05	
SCPL	12.27	2.79	4.45E-5	
Executive functions				
Intercept	-4.45	1.37	0.002	0.10
SCPR	7.13	2.57	0.007	
IPS				
Intercept	-10.75	2.22	9.93E-6	0.31
Left VI	11.54	4.83	0.02	
Right VIIIa	11.15	4.45	0.02	
SCPR	9.03	4.07	0.03	

**Table 7:** Multivariate analysis model 2 –Cognitive prediction and MD analysis.

	Estimate (B)	Standard Error	P value	Adjusted R2
Attention				
Intercept	1.45	0.58	0.02	0.11
Right Crus I	-1635.62	570.94	0.006	
Working Memory				
Intercept	3.75	1.03	0.0006	0.21
Vermis CrusII	-1660.84	804.76	0.04	
SCPR	6237.24	2532.75	0.02	
SCPL	-7812.16	2562.06	0.003	
Executive functions				
Intercept	5.61	1.63	0.001	0.18
ICPL	-5927.83	1540.53	0.0003	
IPS				
Intercept	1.16E1	2.92	0.0002	0.27
Age	-4.75E-2	1.52E-2	0.003	
MCP	-1.02.E4	2.63E3	0.0003	

## Discussion

Our study highlights that microstructural alterations in the posterior cerebellar substructures are associated with impairment in different cognitive domains in MS.

### Microstructural alterations at different stages of MS

We observed that microstructural damages are detected by DTI in posterior lobules and cerebellar peduncles in the MS subgroup but not in CIS. A post-mortem study found an association in MS between DTI metrics (FA and MD) and myelin content and axonal count or gliosis to a lesser proportion, suggesting that DTI is a reliable method for analysing microstructural damage in this disease [38]. However, depending on the DTI-metrics profiles observed, several types of alterations should be discussed.

Concordant with studies in supratentorial brain [17–19,39,40], FA was reduced in most of the cerebellar substructures in CIPwMS compared with PwCIS and HS. Change in MD could be observed in concordance or not with this FA reduction. MD increased in cerebellar peduncles, concordant with FA reduction. This concordant profile is the most frequent when analysing structured bundles of white matter and is associated with fiber loss and alteration of structural barriers limiting water molecular motion. [41]. By contrast, FA decrease was associated with MD preservation in the left VI, left and right Crus I and FA preservation with MD decrease in the vermis Crus II, left VIIb and left and right VIIIb. This discordance has previously been shown in other diseases (post-lacunar Wallerian degeneration and thalamic microstructural changes in Parkinson disease) but is not fully understood [42,43]. FA decrease associated with unmodified MD level is considered to be the consequence of a secondary Wallerian degeneration with axonal loss and changes in neurons integrity accompanied by gliosis or extracellular matrix modifications [42,43]. This process may occur preferentially in regions where fibre tracts are crossing and cellularity is higher than it is in WM. Moreover, an isolated MD upholding could occur when fibre loss is associated with an insufficient cellular reaction to alter directional anisotropy metrics. It has also been observed that glial proliferation could decrease both MD and FA, highlighting a preponderance of tissue damage over tissue repair [41].

In our cohort, FA in CIS tended to rise in comparison to HS. DTI studies in CIS yielded contradictory results, showing either an increase or a decrease of FA in WM and GM according to previous studies [44–46]. This phenomenon has already been described within structures including GM in MS and could be related to the stripping of selective dendrites or iron accumulation [47–50]. More recently, in post-mortem samples undergoing DTI imaging and histological study, this phenomenon has been associated with tissue compaction related to neurodegeneration rather than microglial activation [51].

### Involvement in cognitive impairment

Several DTI studies have focused on the peduncle abnormalities which are now rather well defined in contrary to lobular ones. An association between cognitive impairment (especially IPS assessed by the SDMT and verbal learning) and abnormal superior cerebellar peduncles diffusion parameters has been reported [52]. Reduced FA was also found in cerebellar parenchyma in cognitively impaired patients [19]. Our results showed that cognitive z scores were partly predicted by microstructural alteration in cerebellar peduncles contributing to the disconnection between the supra-tentorial associative areas and the cerebellum. This confirmed the important role of the peduncles in cognitive processes and above all IPS and working memory. Although some researchers studied the correlation between microstructural damages and CIAMS, none have analysed the particular involvement of the cerebellum sub-structures. Interestingly and contrary to some fMRI studies in HS [24,25], we found no evidence of a strict cognitive map arrangement that would link a specific lobule to a cognitive domain. Indeed, multivariate analysis demonstrated that the domain z score could be predicted by different substructure alterations depending on the diffusion parameter that is considered. For example, the attention z score was only correlated with MD in the right Crus I, working memory with FA in the left lobule VI and MD in the vermal Crus II and IPS was strongly correlated with FA in the left lobule VI and right VIIIa.

These results echo the study that we recently reported about GM volumetric analysis in the same group of patients [53]. In that previous work, we showed a correlation between GM volume within posterior lobules and especially vermis VI and IPS. These results showed that both macro and microstructural damages, especially in lobules VI, are associated with cognitive impairment in MS. However, the wider range of structures for which microstructural abnormalities were associated with cognitive deficits suggest that DTI could detect early processes before the development of atrophy.

Our results corroborate fMRI studies showing that working memory should be supported by the lobules VI and VII, for example [25,54]. However, an overlap between working memory and IPS is demonstrated for left lobule VI, indicating a non-formal cognitive map in the posterior cerebellum. The lack of strict mapping is in agreement with Schmahmann’s assumption that the posterior cerebellum “regulates the speed, capacity, consistency, and appropriateness of mental or cognitive processes” [8,9,55] Indeed, the main role of this anatomical structure is to generate an automatized response from high level cognitive load processed in the cortical associative areas. It has been shown that MS patients are unable to activate the usual cerebello-frontal network associated with fastest responses to a given task, consequently activating a substitute compensatory network, involving the prefrontal cortex [23]. IPS represents best this preferential phenomenon of global optimisation and automation which has been previously highlighted by clinical and fMRI studies in MS. [12,23].

### Study limitations

Our study is not without limitations. First, infratentorial lesions have not been considered, although both grey and white matter lesions in the cerebellum could have an impact on cortico-cerebellar disconnection [56]. However, lesions impact DTI metrics, and their effect is therefore included within the variables. Second, in our analysis, ROIs included both grey and white matter. Therefore, the average diffusivity parameters reflected a nonlinear heterogeneous cerebellar anatomy with a risk of statistical bias. Indeed, cerebellar diffusion-weighted imaging is even more challenging than anatomical imaging because of the technical difficulties and anatomical heterogeneity (principally grey and white matter tangles). Volume atrophy leading to DTI parameters modification is another concern. Indeed, cerebellar atrophy was not taken into account in our analysis. Moreover, PwCIS and PwMS were included from two different studies and some tests used for neuropsychological assessment were different. However, the majority of cognitive domains were assessed in the same way between PwCIS and PwMS and divergent tests were roughly equivalent and were always associated to a common test in order to obtain relevant cognitive domains.

## Conclusion

In conclusion, we report the predictive value of DTI metrics in posterior cerebellar lobules and peduncles in cognitive outcome at different stages of MS. IPS and working memory seemed to be more significantly impacted than executive functions and attention, corroborating the idea of cerebellar cognitive regulation and optimization through the cortico-cerebellar loop rather than a cognitive substrate *per se*.

## References

[1] Mori S, Zhang J. Principles of diffusion tensor imaging and its applications to basic neuroscience research. Neuron. 2006; 51: 527–39. doi: 10.1016/j.neuron.2006.08.012 16950152 10.1016/j.neuron.2006.08.012

[2] Rocca MA, Amato MP, De Stefano N, Enzinger C, Geurts JJ, Penner I-K, et al Clinical and imaging assessment of cognitive dysfunction in multiple sclerosis. Lancet Neurol. 2015; 14: 302–17. doi: 10.1016/S1474-4422(14)70250-9 25662900 10.1016/S1474-4422(14)70250-9

[3] Chiaravalloti ND, DeLuca J. Cognitive impairment in multiple sclerosis. Lancet Neurol. 2008; 7: 1139–51. doi: 10.1016/S1474-4422(08)70259-X 19007738 10.1016/S1474-4422(08)70259-X

[4] Ruet A, Deloire M, Charré-Morin J, Hamel D, Brochet B. Cognitive impairment differs between primary progressive and relapsing-remitting MS. Neurology. 2013; 80: 1501–8. doi: 10.1212/WNL.0b013e31828cf82f 23516324 10.1212/WNL.0b013e31828cf82f

[5] Deloire MSA, Salort E, Bonnet M, Arimone Y, Boudineau M, Amieva H, et al Cognitive impairment as marker of diffuse brain abnormalities in early relapsing remitting multiple sclerosis. J Neurol Neurosurg Psychiatry. 2005 4;76(4):519–26. doi: 10.1136/jnnp.2004.045872 15774439 10.1136/jnnp.2004.045872 PMC1739602

[6] Audoin B, Guye M, Reuter F, Au Duong M-V, Confort-Gouny S, Malikova I, et al Structure of WM bundles constituting the working memory system in early multiple sclerosis: a quantitative DTI tractography study. NeuroImage. 2007; 36: 1324–30. doi: 10.1016/j.neuroimage.2007.04.038 17513134 10.1016/j.neuroimage.2007.04.038

[7] Štecková T, Hluštík P, Sládková V, Odstrčil F, Mareš J, Kaňovský P. Thalamic atrophy and cognitive impairment in clinically isolated syndrome and multiple sclerosis. J Neurol Sci. 2014; 342: 62–8. doi: 10.1016/j.jns.2014.04.026 24819917 10.1016/j.jns.2014.04.026

[8] Schmahmann JD. Dysmetria of thought: clinical consequences of cerebellar dysfunction on cognition and affect. Trends Cogn Sci. 1998;2:362–71. 21227233 10.1016/s1364-6613(98)01218-2

[9] Schmahmann JD, Caplan D. Cognition, emotion and the cerebellum. Brain. 2006; 129: 290–2. doi: 10.1093/brain/awh729 16434422 10.1093/brain/awh729

[10] Valentino P, Cerasa A, Chiriaco C, Nisticò R, Pirritano D, Gioia M, et al Cognitive deficits in multiple sclerosis patients with cerebellar symptoms. Mult Sclerl. 2009; 15: 854–9. 10.1177/1352458509104589 19542264

[11] Weier K, Penner IK, Magon S, Amann M, Naegelin Y, Andelova M, et al Cerebellar abnormalities contribute to disability including cognitive impairment in multiple sclerosis. PloS One. 2014; 9: e86916 doi: 10.1371/journal.pone.0086916 24466290 10.1371/journal.pone.0086916 PMC3899307

[12] Ruet A, Hamel D, Deloire MSA, Charré-Morin J, Saubusse A, Brochet B. Information processing speed impairment and cerebellar dysfunction in relapsing-remitting multiple sclerosis. J Neurol Sci. 2014; 347: 246–50. doi: 10.1016/j.jns.2014.10.008 25454642 10.1016/j.jns.2014.10.008

[13] Kutzelnigg A, Faber-Rod JC, Bauer J, Lucchinetti CF, Sorensen PS, Laursen H, et al Widespread demyelination in the cerebellar cortex in multiple sclerosis. Brain Pathol. 2007; 17: 38–44. doi: 10.1111/j.1750-3639.2006.00041.x 17493036 10.1111/j.1750-3639.2006.00041.x PMC8095596

[14] Morgen K, Sammer G, Courtney SM, Wolters T, Melchior H, Blecker CR, et al Evidence for a direct association between cortical atrophy and cognitive impairment in relapsing–remitting MS. NeuroImage. 2006; 30: 891–8. doi: 10.1016/j.neuroimage.2005.10.032 16360321 10.1016/j.neuroimage.2005.10.032

[15] van de Pavert SHP, Muhlert N, Sethi V, Wheeler-Kingshott CAM, Ridgway GR, Geurts JJG, et al DIR-visible grey matter lesions and atrophy in multiple sclerosis: partners in crime? J Neurol Neurosurg Psychiatry. 2016; 87: 461–7. doi: 10.1136/jnnp-2014-310142 25926483 10.1136/jnnp-2014-310142 PMC4853554

[16] Weier K, Till C, Fonov V, Yeh EA, Arnold DL, Banwell B, et al Contribution of the cerebellum to cognitive performance in children and adolescents with multiple sclerosis. Mult Scler. 2016 22; 599–607. doi: 10.1177/1352458515595132 26203072 10.1177/1352458515595132

[17] Mesaros S, Rocca MA, Kacar K, Kostic J, Copetti M, Stosic-Opincal T, et al Diffusion tensor MRI tractography and cognitive impairment in multiple sclerosis. Neurology. 2012; 78: 969–75. doi: 10.1212/WNL.0b013e31824d5859 22377806 10.1212/WNL.0b013e31824d5859

[18] Yu HJ, Christodoulou C, Bhise V, Greenblatt D, Patel Y, Serafin D, et al Multiple white matter tract abnormalities underlie cognitive impairment in RRMS. NeuroImage. 2012; 59: 3713–22. doi: 10.1016/j.neuroimage.2011.10.053 22062194 10.1016/j.neuroimage.2011.10.053

[19] Hulst HE, Steenwijk MD, Versteeg A, Pouwels PJW, Vrenken H, Uitdehaag BMJ, et al Cognitive impairment in MS: impact of white matter integrity, gray matter volume, and lesions. Neurology. 2013; 80: 1025–32. doi: 10.1212/WNL.0b013e31828726cc 23468546 10.1212/WNL.0b013e31828726cc

[20] Sbardella E, Petsas N, Tona F, Prosperini L, Raz E, Pace G, et al Assessing the correlation between grey and white matter damage with motor and cognitive impairment in multiple sclerosis patients. PloS One. 2013; 8: e63250 doi: 10.1371/journal.pone.0063250 23696802 10.1371/journal.pone.0063250 PMC3655958

[21] Kolbe SC, Kilpatrick TJ, Mitchell PJ, White O, Egan GF, Fielding J. Inhibitory saccadic dysfunction is associated with cerebellar injury in multiple sclerosis. Hum Brain Mapp. 2014; 35: 2310–9. doi: 10.1002/hbm.22329 24038970 10.1002/hbm.22329 PMC6869843

[22] Bozzali M, Spanò B, Parker GJM, Giulietti G, Castelli M, Basile B, et al Anatomical brain connectivity can assess cognitive dysfunction in multiple sclerosis. Mult Scler. 2013; 19: 1161–8. doi: 10.1177/1352458512474088 23325589 10.1177/1352458512474088

[23] Bonnet MC, Allard M, Dilharreguy B, Deloire M, Petry KG, Brochet B. Cognitive compensation failure in multiple sclerosis. Neurology. 2010; 75: 1241–8. doi: 10.1212/WNL.0b013e3181f612e3 20921510 10.1212/WNL.0b013e3181f612e3

[24] Keren-Happuch E, Chen S-HA, Ho M-HR, Desmond JE. A Meta-analysis of Cerebellar Contributions to Higher Cognition from PET and fMRI studies. Hum Brain Mapp. 2014; 35: 593–615. doi: 10.1002/hbm.22194 23125108 10.1002/hbm.22194 PMC3866223

[25] Stoodley CJ, Schmahmann J D. Evidence for topographic organization in the cerebellum of motor control versus cognitive and affective processing. Cortex. 2010;46:831–844. doi: 10.1016/j.cortex.2009.11.008 20152963 10.1016/j.cortex.2009.11.008 PMC2873095

[26] Schmahmann JD, Pandya D N. Anatomic organization of the basilar pontine projections from prefrontal cortices in rhesus monkey. J Neurosci. 1997; 17: 438–458. 8987769 10.1523/JNEUROSCI.17-01-00438.1997 PMC6793685

[27] Middleton FA, Strick PL. Cerebellar projections to the prefrontal cortex of the primate. J Neurosci. 2001;21: 700–712. 11160449 10.1523/JNEUROSCI.21-02-00700.2001 PMC6763818

[28] Allen G, Buxton R B, Wong EC, Courchesne E. Attentional Activation of the Cerebellum Independent of Motor Involvement. Science. 1997; 275: 1940–1943. 9072973 10.1126/science.275.5308.1940

[29] Polman CH, Reingold SC, Edan G, Filippi M, Hartung H-P, Kappos L, et al Diagnostic criteria for multiple sclerosis: 2005 revisions to the ‘McDonald Criteria’. Ann Neurol. 2005; 58: 840–6. doi: 10.1002/ana.20703 16283615 10.1002/ana.20703

[30] Lamargue-Hamel D, Deloire M, Saubusse A, Ruet A, Taillard J, Philip P, et al Cognitive evaluation by tasks in a virtual reality environment in multiple sclerosis. J Neurol Sci. 2015; 359: 94–9. doi: 10.1016/j.jns.2015.10.039 26671094 10.1016/j.jns.2015.10.039

[31] Manjón JV, Coupé P, Martí-Bonmatí L, Collins DL, Robles M. Adaptive non-local means denoising of MR images with spatially varying noise levels. J Magn Reson Imaging JMRI. 2010; 31: 192–203. doi: 10.1002/jmri.22003 20027588 10.1002/jmri.22003

[32] Avants BB, Tustison NJ, Song G, Cook PA, Klein A, Gee JC. A reproducible evaluation of ANTs similarity metric performance in brain image registration. NeuroImage. 2011; 54: 2033–44. doi: 10.1016/j.neuroimage.2010.09.025 20851191 10.1016/j.neuroimage.2010.09.025 PMC3065962

[33] Manjón JV, Coupé P, Concha L, Buades A, Collins DL, Robles M. Diffusion weighted image denoising using overcomplete local PCA. PLoS One. 2013; 8: e73021 doi: 10.1371/journal.pone.0073021 24019889 10.1371/journal.pone.0073021 PMC3760829

[34] http://www.diedrichsenlab.org/imaging/suit.htm.

[35] Diedrichsen J. A spatially unbiased atlas template of the cerebellum and brainstem (SUIT). NeuroImage. 2006;33: 127–38. 16904911 10.1016/j.neuroimage.2006.05.056

[36] Diedrichsen J, Balsters JH, Flavell J, Cussans E, Ramnani N. A probabilistic MR atlas of the human cerebellum. NeuroImage. 2009;46:39–46. doi: 10.1016/j.neuroimage.2009.01.045 19457380 10.1016/j.neuroimage.2009.01.045

[37] Ashburner J. A fast diffeomorphic image registration algorithm. NeuroImage. 2007;38:95–113. doi: 10.1016/j.neuroimage.2007.07.007 17761438 10.1016/j.neuroimage.2007.07.007

[38] Schmierer K, Wheeler-Kingshott CAM, Boulby PA, Scaravilli F, Altmann DR, Barker GJ, et al Diffusion tensor imaging of post mortem multiple sclerosis brain. Neuroimage. 2007; 35: 467–77. doi: 10.1016/j.neuroimage.2006.12.010 17258908 10.1016/j.neuroimage.2006.12.010 PMC1892244

[39] Dineen RA, Vilisaar J, Hlinka J, Bradshaw CM, Morgan PS, Constantinescu CS, et al Disconnection as a mechanism for cognitive dysfunction in multiple sclerosis. Brain. 2009; 132: 239–49. doi: 10.1093/brain/awn275 18953055 10.1093/brain/awn275

[40] Roca M, Torralva T, Meli F, Fiol M, Calcagno ML, Carpintiero S, et al Cognitive deficits in multiple sclerosis correlate with changes in fronto-subcortical tracts. Mult Scler. 2008; 14: 364–9. doi: 10.1177/1352458507084270 18208880 10.1177/1352458507084270

[41] Rovaris M, Gass A, Bammer R, Hickman SJ, Ciccarelli O, Miller DH, et al Diffusion MRI in multiple sclerosis. Neurology. 2005; 65: 1526–32. doi: 10.1212/01.wnl.0000184471.83948.e0 16301477 10.1212/01.wnl.0000184471.83948.e0

[42] Pierpaoli C, Barnett A, Pajevic S, Chen R, Penix L, Virta A, et al Water Diffusion Changes in Wallerian Degeneration and Their Dependence on White Matter Architecture. NeuroImage. 2001; 13: 1174–85. doi: 10.1006/nimg.2001.0765 11352623 10.1006/nimg.2001.0765

[43] Li W, Liu J, Skidmore F, Liu Y, Tian J, Li K. White matter microstructure changes in the thalamus in Parkinson disease with depression: A diffusion tensor MR imaging study. AJNR Am J Neuroradiol. 2010;31:1861–6. doi: 10.3174/ajnr.A2195 20705702 10.3174/ajnr.A2195 PMC7964038

[44] Cappellani R, Bergsland N, Weinstock-Guttman B, Kennedy C, Carl E, Ramasamy DP, et al Diffusion tensor MRI alterations of subcortical deep gray matter in clinically isolated syndrome. J Neurol Sci. 2014; 338: 128–34. doi: 10.1016/j.jns.2013.12.031 24423584 10.1016/j.jns.2013.12.031

[45] Gallo A, Rovaris M, Riva R, et al DIffusion-tensor magnetic resonance imaging detects normal-appearing white matter damage unrelated to short-term disease activity in patients at the earliest clinical stage of multiple sclerosis. Arch Neurol. 2005; 62: 803–8. doi: 10.1001/archneur.62.5.803 15883269 10.1001/archneur.62.5.803

[46] Caramia F, Pantano P, Di Legge S, Piattella MC, Lenzi D, Paolillo A, et al A longitudinal study of MR diffusion changes in normal appearing white matter of patients with early multiple sclerosis. Magn Reson Imaging. 2002; 20: 383–8. 12206862 10.1016/s0730-725x(02)00519-2

[47] Ciccarelli O, Werring DJ, Wheeler-Kingshott CA, Barker GJ, Parker GJ, Thompson AJ, et al Investigation of MS normal-appearing brain using diffusion tensor MRI with clinical correlations. Neurology. 2001; 56: 926–33. 11294931 10.1212/wnl.56.7.926

[48] Tovar-Moll F, Evangelou IE, Chiu AW, Richert ND, Ostuni JL, Ohayon JM, et al Thalamic Involvement and Its Impact on Clinical Disability in Patients with Multiple Sclerosis: A Diffusion Tensor Imaging Study at 3T. Am J Neuroradiol. 2009; 30: 1380–6. doi: 10.3174/ajnr.A1564 19369608 10.3174/ajnr.A1564 PMC7051560

[49] Hannoun S, Durand-Dubief F, Confavreux C, Ibarrola D, Streichenberger N, Cotton F, et al Diffusion tensor-MRI evidence for extra-axonal neuronal degeneration in caudate and thalamic nuclei of patients with multiple sclerosis. AJNR Am J Neuroradiol. 2012; 33: 1363–8. doi: 10.3174/ajnr.A2983 22383236 10.3174/ajnr.A2983 PMC7965527

[50] Cavallari M, Ceccarelli A, Wang G-Y, Moscufo N, Hannoun S, Matulis CR, et al Microstructural changes in the striatum and their impact on motor and neuropsychological performance in patients with multiple sclerosis. PloS One. 2014; 9: e101199 doi: 10.1371/journal.pone.0101199 25047083 10.1371/journal.pone.0101199 PMC4105540

[51] Jonkman LE, Klaver R, Fleysher L, Inglese M, Geurts JJ. The substrate of increased cortical FA in MS: A 7T post-mortem MRI and histopathology study. Mult Scler. 2016 3 4 pii: 1352458516635290. [Epub ahead of print] doi: 10.1177/1352458516635290 . 26945031 10.1177/1352458516635290

[52] Preziosa P, Rocca MA, Mesaros S, Pagani E, Drulovic J, Stosic-Opincal T, et al Relationship between Damage to the Cerebellar Peduncles and Clinical Disability in Multiple Sclerosis. Radiology. 2014;271:822–830. doi: 10.1148/radiol.13132142 24555637 10.1148/radiol.13132142

[53] Moroso A, Ruet A, Lamargue-Hamel D, Munsch F, Deloire M, Coupé P, et al Posterior lobules of the cerebellum and information processing speed at various stages of multiple sclerosis. J Neurol Neurosurg Psychiatry. 2017;88:146–151 doi: 10.1136/jnnp-2016-313867 27789541 10.1136/jnnp-2016-313867

[54] Hayter AL, Langdon DW, Ramnani N. Cerebellar contributions to working memory. NeuroImage. 2007; 36: 943–54. doi: 10.1016/j.neuroimage.2007.03.011 17468013 10.1016/j.neuroimage.2007.03.011

[55] Schmahmann JD. From movement to thought: anatomic substrates of the cerebellar contribution to cognitive processing. Hum Brain Mapp. 1996; 4: 174–98. doi: 10.1002/(SICI)1097-0193(1996)4:3<174::AID-HBM3>3.0.CO;2-0 20408197 10.1002/(SICI)1097-0193(1996)4:3<174::AID-HBM3>3.0.CO;2-0

[56] Clausi S, Bozzali M, Leggio MG, Di Paola M, Hagberg GE, Caltagirone C, et al Quantification of gray matter changes in the cerebral cortex after isolated cerebellar damage: a voxel-based morphometry study. Neuroscience. 2009; 162: 827–35. doi: 10.1016/j.neuroscience.2009.02.001 19409211 10.1016/j.neuroscience.2009.02.001

